# Major Molecular Factors Related to *Leishmania* Pathogenicity

**DOI:** 10.3389/fimmu.2022.847797

**Published:** 2022-06-13

**Authors:** Hanan S. Al-Khalaifah

**Affiliations:** Environment and Life Sciences Research Center, Kuwait Institute for Scientific Research, Kuwait City, Kuwait

**Keywords:** anti-parasite drugs, macrophages, leishmaniasis, pathoantigenic determinants, infective

## Abstract

Leishmaniasis is a major health problem with 600k - 1M new cases worldwide and 1 billion at risk. It involves a wide range of clinical forms ranging from self-healing cutaneous lesions to systemic diseases that are fatal if not treated, depending on the species of *Leishmania. Leishmania* sp. are digenetic parasites that have two different morphological stages. *Leishmania* parasites possess a number of invasive/evasive and pathoantigenic determinants that seem to have critical roles in *Leishmania* infection of macrophages which leads to successful intracellular parasitism in the parasitophorous vacuoles. These determinants are traditionally known as “virulence factors”, and are considered to be good targets for developing specific inhibitors to attenuate virulence of *Leishmania* by gene deletions or modifications, thus causing infective, but non-pathogenic mutants for vaccination. Pathway of biosynthesis is critical for keeping the parasite viable and is important for drug designing against these parasites. These drugs are aimed to target enzymes that control these pathways. Accordingly, maintaining low level of parasitic infection and in some cases as a weapon to eradicate infection completely. The current paper focuses on several virulence factors as determinants of *Leishmania* pathogenicity, as well as the metabolites produced by *Leishmania* to secure its survival in the host.

## Introduction

### Clinical Forms of Leishmaniasis

Leishmaniasis is a serious infectious disease that infects a wide-ranging vertebrates throughout the developing world. It involves a widespread clinical forms ranging from self-healing cutaneous lesions to systemic diseases that are fatal if not treated, depending on the species of *Leishmania.* It is a major health problem as World Health Organization (WHO) estimated 600k - 1M new leishmaniasis cases worldwide and 1 billion at risk. There are three main types of the disease; the cutaneous leishmaniasis which is the utmost popular form. It begins with a small skin lesion of around 1 cm that increases in size. In most of the cases, when healing occurs, there is 100% immunity against re-infection. In some individuals, failure in cell-mediated immunity causes leishmaniasis diffusa that covers most of the skin surface, just like lepromatous leprosy (1, 2). The second major form of the disease is the mucocutaneous leishmaniasis where there is permanent destruction of the mucous membrane in the mouth, nose and throat cavities. The third main form is the visceral leishmaniasis that is considered to be the most severe form. It is caused by *L. donovani, L. infantum*, and *L. infantum chagasi*. This form is fatal if not treated and usually infects the spleen, the liver and the bone marrow. In 20% of the treated patients, a hypo- pigmented skin rash develops after 6 months or more, usually in the face and the upper parts of the body. This condition is called post Kala-azar dermal leishmaniasis (3–6).

### Molecular Determinants of *Leishmania* Virulence

Interestingly, *Leishmania* parasites are able to initiate intracellular parasitism in the parasitophorous vacuoles of the macrophages (7). The key elements that determine parasitism and degree of pathogenicity are mainly molecular determinants of the parasite. These molecules are traditionally called virulence factors. Interactions of these factors determine the degree of pathogenicity (virulence) that is measured as the parasitemia level and/or lesion size (1, 8, 9). These factors enable the parasite to pre-adapt to the mammalian host increased temperature and decreased pH inside the macrophages, required for the initial establishment at the bite site, required for macrophage invasion and for proliferation within the extreme conditions in the phagolysosomal compartments, and are used to avoid the cellular and humoral immune attack of the host (10). It is important to note that these determinants are not direct causative agents of the clinical symptoms of leishmaniasis; this is supported by the fact that direct injection of some virulence factors (e.g. LPG, lipophosphoglycan) into susceptible animals does not cause typical leishmaniasis (5). Since the drugs currently used for leishmaniasis treatment are limited by price, and safety, it is critical to know how the parasite is defending its self in the host cell in order to approach suitable therapeutic treatment.

**Figure 1:**
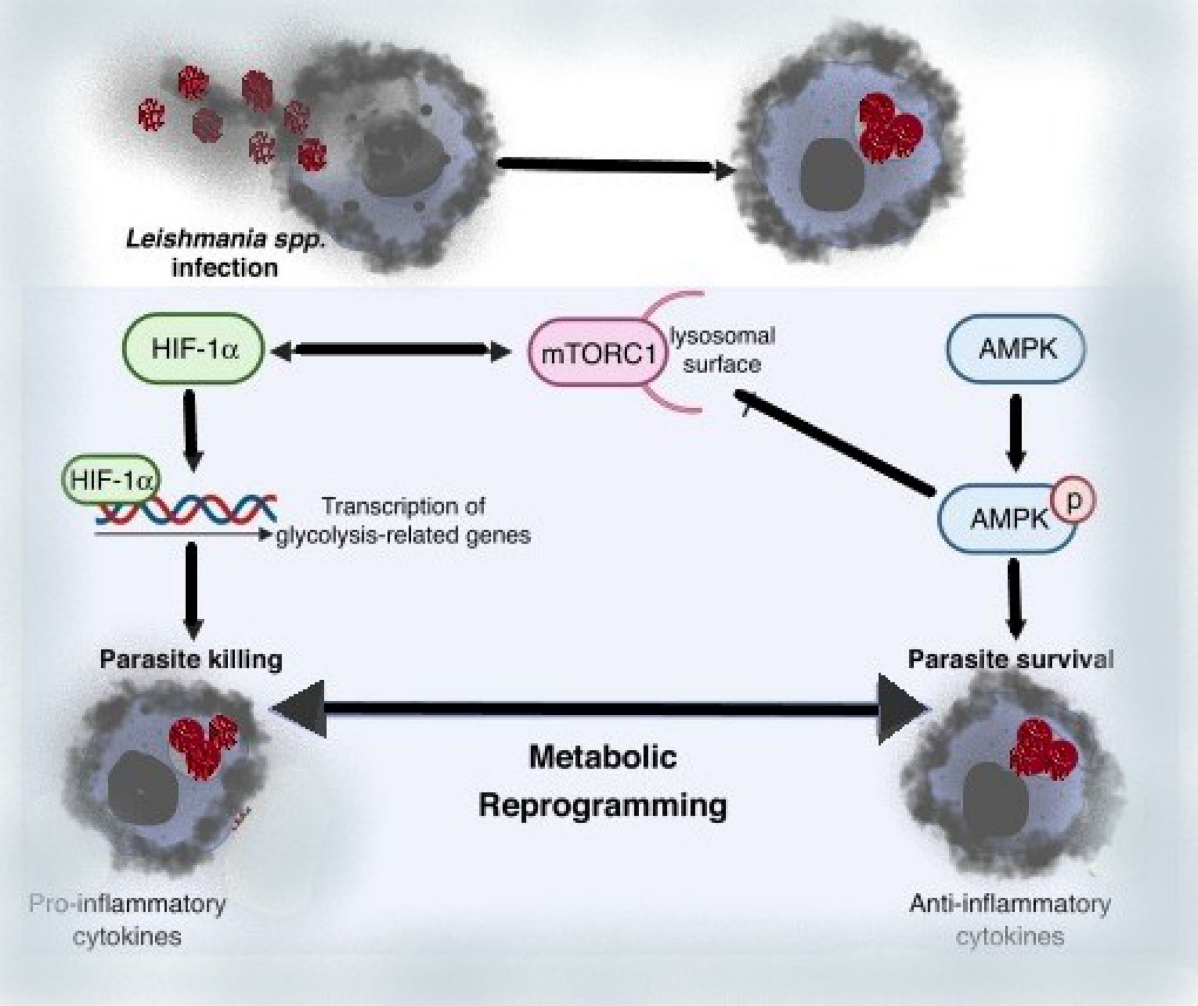
Virulence factors of Leishmania.

Elmahallawy and Alkhaldi (11) concluded that *Leishmania* can persist in host cells through influencing the host’s immune system in a variety of ways, including causing immunosuppression and changing the host’s chemokine patterns. Leishmaniasis pathogenesis varies widely depending on a variety of factors, including the infecting species and its virulence factors, as well as the host, all of which influence the disease’s outcome (**Figure 1**).

### Superoxide Dismutase

Superoxide dismutase is considered the first line of defence by the parasite by combining superoxide radicals to form molecular oxygen and hydrogen peroxide. This is followed by degradation of the peroxide by catalases or peroxidases to oxygen and water. Inactivation of the toxic peroxidases is catalyzed by catalase enzyme (12).

### Trypanothione Reductase

Studies have revealed that trypanothione reductase, the enzyme that maintain trypanothione in its reduced form, is important for the parasite to stay alive against the oxidative stress inside the macrophages (13). Knock-out mutants by gene disruption in *L.donovani* and *L.major* strains by means of the selectable markers neomycin and hygromycin phosphotransferases show weakened infectivity and a reduced survival capacity in the macrophages (14). Tunes, Morato (15) revealed that using gold complexes can act against *L. infantum* and *L. braziliensis* intracellular amastigotes by causing mitochondrial damage and oxidative stress due to creation of reactive oxygen species. The author used BALB/c mice infected with luciferase-expressing L. braziliensis or L. amazonensis. These mice were treated with oral administration of 12.5 mg/kg/day of AdT Et or AdO Et. Bio-imaging revealed decreased lesion size and parasite burden.

### Glycoconjugates

The parasite surface is mainly occupied by glycoconjugates. The major sugar component of these molecules is mannose. Their abundance and the uniqueness of their chemistry suggest important roles in the parasites virulence and pathogenesis (16, 17). *Leishmania* possess a variety of glycoconjugates that are essential for parasite virulence and pathogenesis. Of these: lipophosphoglycan or LPG, proteins with GPI anchors such as gp63, a smaller group of glycoinositol phospholipids or GIPLs, and proteophosphoglycans or PPG. In *Leishmania*, Manα1-4GlcN-PI is mainly shared between these glycoconjugates, but glycan parts, lipid moieties, and phospholipid precursors are different in the glycoconjugates (18, 19).

### Lipophosphoglycan

These are one of the most important glycoconjugates at the exterior of *Leishmania* species. It is highly expressed in the promastigotes and very little if any is expressed in the intracellular amastigote form. It consists of 15–30 repeating units of phosphorylated oligosaccharides that are linearly connected by phosphodiester bounds. It can be substituted with other sugars depending on the species and is terminated by a capping oligosaccharide. The terminal part of the LPG is coated with a neutral oligosaccharide (19). Although LPG fragments are well-maintained, there are species-specific alterations in the repeated subdivisions. Studies have shown that LPG construction is altered throughout metacyclogenesis and differentiation of *L. major* promastigotes from a less contagious form in the logarithmic growth phase to a greatly contagious form throughout the stationary growth phase (19). During metacyclogenesis, the normal numeral of the repeated fragments is folded from 14 to 30. Also, the repeated fragments with side chains of beta Gal or Gal beta 1-3Gal beta 1- are decreased in number while the repeat units with side chains of Arap alpha 1-2 Gal beta 1- are increased (19).

LPG mediates the binding of promastigotes to the epithelial cells of the sandfly’s midgut, protects *Leishmania* during the blood meal digestion in the midgut of the sandfly, acts as acceptor of C3 complement component, inhibits cell signaling for resisting oxidative burst, interferes with the signaling pathway of protein kinase, and inhibits cytokine production in the macrophages (19–21).

In order for a gene to be a virulence gene, it should fulfill the principles of Koch’s postulates on the molecular level. It must be associated with infectivity and pathogenesis. Also, inactivation of the gene must cause loss of virulence. Finally, re-expression of the gene must restore pathogenesis (22). In the case of *Leishmania*, homozygous null mutants are created by knocking out both of the alleles to observe the resulting phenotype. This is considered to be a limitation if this gene is required for viability. According to these principles, LPG gene is a virulence gene because *lpg1^_^* mutants (i.e. do not have LPG gene) produced by homozygous disruption of genes in the instance of *L. major* were not able to bind to the sand-fly midgut and they didn’t survive after a blood meal digestion while *lpg^+^* types do (i.e. do have LPG gene). Colonies of *Phlebotomus papatasi* were infected with lpg1- *L. major* mutants. The mutant parasites persisted and propagated customarily in the fly midgut but they were vanished from the gut more speedily than the wild type parasites after blood meals. According, LPG is not vital for existence of *L. major* in the early stage of blood-feeding but it is vital to facilitate midgut anchoring and to sustain contagion in the midgut throughout the process of blood digestion. The promastigotes that already invade the macrophages were eliminated in 2 days. Restoration of the LPG gene restored virulence and the amastigotes were able to proliferate in the macrophages. Studies have also revealed that inoculation of 106 promastigotes into the footpad of BALB/c mice (experimental mice) created a wound by day 15 and caused death in due time. On the other hand, inoculation of 106 *lpg-* parasites revealed delayed wound development and again, restoration of the LPG gene restored virulence (21). A similar study also revealed the same results where *Leishmania major lpg1* ^-^ mutants showed reduced virulence and were greatly vulnerable to human complement lysis system (16, 19, 23, 24).

Surprisingly, there was no loss of virulence in the case of *L. mexicana.* LPG deficient parasites continue to be contagious to macrophages and BALB/c mice. So, LPG is not a virulence factor in case of *L. Mexicana* (21, 25).

In addition to LPG as a virulence factor, another major surface glycoprotein is a GPI-anchored (glycosyl phosphatidylinositol anchors) zinc metalloprotease of 63 kD. This molecule is usually called GP63 and alternatively called leishmanolysin or major surface protein (MSP). It is the utmost present glycoprotein in *Leishmania* species. In *L. mexicana*, there are approximately 5X10^5^ MSP molecules. All *Leishmania* sp. inspected have numerous tandem genes encoding gp63 (26, 27). Gp63 in ten *Leishmania* sp. occurs in both amphiphilic and hydrophilic forms, encoding the same amino acid sequences. It consists of a predicted protein sequence containing the N-terminal hydrophobic sequence and a pro-peptide that is detached upon development (28), the later sequence involves a conserved cysteine residue that is shown in *L. major* to be critical in protecting the parasite from self-destruction due to active protease activity. Differences in gp63 structure among *Leishmania* sp. include differences in the C-terminal sequence, the 3’ untranslated sequence, and the differential expression in different life stages (27, 29). It is present on surfaces of both promastigotes and amastigotes and has a great role in degrading a range of protein substances and in facilitating attachment to macrophages by acting as opsonin (29). In addition, it inhibits complement-mediated lysis by binding to the complement component C3. It then converts the active C3b molecules into the inactive C3bi (29, 30). Also, GP63 protects the amastigotes from the adverse conditions in the macrophage phagolysosomes; this is evidenced by the point that it was able to protect bovine serum albumin in the same environment. It is suggested that gp63 interferes with immune response of the mammalian host *via* inhibiting antigen presentation on Class I molecules (31). Additionally, it was revealed that gp63 in *L. amazonensis* enhanced degradation of the extracellular matrix and basement membrane proteins; this suggests its importance in establishing the infection and migration of the parasite *via* macrophages circulation to deeper tissues like the spleen and the liver to establish visceral leishmaniasis (32). *In vitro* degradation of extra-cellular matrix constituents such as collagen, fibronectin, and laminin by gp63 supports its responsibility in parasite movement and infection creation.

Inhibition of the parasite activity can be achieved by using anti-gp63 monoclonal antibodies. Knock-out parasites for the genes encoding for the gp63 family in *L. mexicana* showed less virulence than the wild type parasites and were very sensitive to complement mediated lysis. Accordingly, this protein is necessary to support parasite existence. Interestingly, *L. major* gp63- mutants survived and proliferated normally in the macrophages (31, 33). It is interesting to know that expressions of LPG and GP63 are not related. A study has revealed that *LPG1* does not affect the expression of gp63 where crude cell extracts (2.5 X 10^6^ cells) from logarithmic cultures were exposed Western blotting with anti-gp63 antibody. Flow cytometry has also been used for this purpose where the fixed permeabilized parasites are labeled with anti-gp63 antibody. Control parasites were similarly treated except that anti-gp63 antibodies were not used (34).

Interestingly, agglutination experiments using CA7AE antibodies and a lectin were used to select knockout parasites (RCA 120). Five clones were obtained and molecularly analyzed, all of which revealed the expected altered genome as well as the total lack of expression of LPG and PG-containing molecules. Finally, it was discovered that deletion of LPG2 impairs the result of infection in human neutrophils, as evidenced by an 83 percent reduction in intracellular load compared to wild-type parasite infection. The findings support the role of LPG and other PGs in host-parasite interactions as virulence factors (35).

### Cysteine Proteinase

In addition, cysteine proteinase (CP) is considered to be a virulence factor in *Leishmania* sp. It is more expressed in the amastigotes form than the promastigote form. In general, these proteins are believed to have a great role in degrading lysosomal proteases. The cathepsin L-like cysteine proteinases (CPs) of parasitic protozoa are known to influence other vital parasite activities such as nutrition ^52^ and neutralization of the host immune system (36).

Studies have shown that *L.mexicana* has cathepsin L-like cysteine proteinase genes that are a multicopy of 19 genes (*lmcpb*) and two single copy CP genes (*lmcpa and lmcpc*). *Lmcpb* null mutants were able to proliferate and differentiate *in vitro*, however, infectivity to macrophages was decreased by 80%. The mutants created subcutaneous lesions in mice in a rate less than the wild type parasites. Re-expression of a single copy of *lmcpb* restored infectivity (37). The lesions resulting from infection with Δ*cpb* appeared slowly at wk 31 and were very minor (mean lesion volume at wk 37 was 3.5 mm^3^), while injection of Δ*cpa/cpb* did not produce lesions (38). In addition, *L.mexicana* was shown to be sensitive to cysteine proteinase inhibitors *in vitro*, indicating the importance of CP for *Leishmania* survival (37).

Studies have also revealed that the main cysteine protease of *T. cruzi*, cruzain, has been connected to plasma leakage in post-capillary venules and may recruit macrophages for invasion (39).

It was observed that apoptosis (i.e. programmed cell death) in *L. donovani* involves caspase like activity that can be inhibited using cysteine protease inhibitors. Apoptosis in this intracellular parasite regulate population growth during infection and prolong parasite survival in macrophages. In *L. major*, however, cathepsin B-like inhibitors reduced DNA fragmentation but did not influence apoptosis. A recent study has identified a gene coding for a protein with high degree of homology to a mitogen-activated protein (MAP) kinase in *L. mexicana*. A deletion mutant for the gene locus encoding for Secreted Acid Phosphatase (SAP) and containing the intergenic region of ~ 11.5 kb has been prepared. This mutant parasite was not able to produce leishmaniasis in Balb/c mice. However, the infectivity was restored when a 6 kb region of the SAP locus was introduced. This region was shown to contain two Open Reading Frames encoding single copy genes. One of them (*ORF1)* codes for a protein of 358 amino acids of a molecular weight of 41 kDa, this is called LMPK and is considered to be a homologue of the MAP kinase that is essential for *Leishmania* differentiation in the macrophages. It is up-regulated in amastigotes as compared to its expression mRNA levels in promastigotes. Polyclonal anti-serum against the C-terminal peptide of LMPK was raised in rabbits and affinity chromatography was used for purification. Immunoblotting of LMPK from cell lysates of both amastigotes and promastigotes has been done (40, 41).

### Free Glycoinositol Phospholipids

Free glycoinositol phospholipids (GIPLs) molecules have been also found on the surface of the genus *Leishmania*; these are not linked to protein or phosphoglycan anchors and are thought to be virulence factors in *Leishmania* (42, 43). *In vitro* and *in vivo* studies have revealed that GIPLs (and LPG) can modify the action of membrane-associated protein tyrosine kinases and protein kinase C in host cells (43, 44). A study has shown that *L. mexicana* promastigotes synthesize two distinct GIPL lineages, including at least 10 glycolipid species (45). Dolichol-phosphate-mannose synthase (DPMS) is an important enzyme in *Leishmania* sp., because it stimulates the formation of DPM through the transfer of mannose from GDP-Man to dolichol-phosphate. DPM is the only mannose donor for three mannose residues that structure the trimannose backbone in the GPI protein anchor precursors. A study has shown that creation of *L.mexicana* null mutants by directed distraction of both alleles of the gene that encodes DPMS, namely *lmdpms*, caused a augmentation of the chromosomal *lmdpms* locus indicating that this enzyme is critical for growing due to its role in GIPLs biosynthesis and that GIPLs are essential membrane components in *L.mexicana* promastigotes (46, 47). The main role of the GIPLs in *Leishmania* is not very clear. Intracellular partitions containing GIPLs include the megasomes in *L.mexicana* amastigotes. By similarity with the responsibility of glycosphingolipids in animals, these GIPLs may have a role in establishing a defensive layer of glycocalyx to protect lysosomal membranes from luminal enzymes. Also, the GIPLs may have a role as intermediaries in endogenous signal transduction pathways (48, 49).

In addition, protein phosphorylation is very important in order for *Leishmania* to proliferate and differentiate in the macrophages. Recently, it was found that *Leishmania* parasites discharge a range of proteins that are altered by phosphoglycan fragments analogous to those of the surface glycolipid lipophosphoglycans. These proteins are known as proteophosphoglycans or PPG. These elements contains acid phosphatase manufactured by promastigotes of all *Leishmania* sp. except *Leishmania major*, non-filamentous proteophosphoglycan of *Leishmania mexicana* amastigotes, and a filamentous proteophosphoglycan (fPPG) produced by promastigotes of all *Leishmania* sp. Capped phosphoglycan chains are linked to the polypeptide backbone of these proteins *via* phosphodiester linkages to serine (50–52).

This mechanism of phosphorylation involves regulation of protein kinases and phosphatases. Secretory acid phosphatase (SAP) is believed to be an important virulence factor in *Leishmania* species. This protein is secreted from the endoplasmic reticulum then it is transferred to the surface or is secreted *via* the flagellar reservoir (18, 53, 54). Protein Disulfide Isomerase (PDI) of the endoplasmic reticulum plays a critical action in controlling the secretion of acid phosphates. It catalyzes the oxidation and isomerization of protein disulfide linkages in the endoplasmic reticulum. Studies have revealed the presence of a 12 kDa single thioredoxin-like domain containing PDI in *L. donovani*. Over expression of PDI mutants in *L. donovani* considerably reduced the production of acid phosphatase. In *L. major*, it was observed that highly virulent strains of the parasite contain increased expression of PDI, suggesting a role of PDI and secreted acid phosphatase in supporting the parasite survival in the mammalian host (40).

Investigations by immunofluorescence and immunoelectron microscopes on two Leishmania/sandfly vector combinations (*Leishmania mexicana*/*Lutzomyia longipalpis* and *L. major*/*Phlebotomus papatasi*) has revealed the presence of a dense three-dimensional network of filaments that surrounds the promastigote cell bodies in a gel-like mass formed mainly by a parasite-derived mucin-like filamentous proteophosphoglycan (fPPG). Accordingly, it was proposed that the constant discharge of fPPG by promastigotes in the sandfly gut is an important factor in an efficient transmission of the parasite to the mammalian host (55). The fPPG gene has been cloned by antibody screening of a *L. major* genomic expression library, leading to the documentation of repetitive DNA fragments that encode for Ser, Ala, and Pro in ratios in line with the known configuration of fPPG (56).

The non-filamentous proteophosphoglycan were shown to be secreted from the intracellular amastigote form of the parasitic protozoon *Leishmania mexicana*. This high-molecular weight phosphoglycan was purified from a cell-free homogenate of infected mouse tissue and from amastigotes and was shown to consist of serine-rich polypeptide chains and mild acid-labile phosphooligosaccharides capped by mannooligosaccharides. Immunofluorescence and immune-electron microscopy studies suggest that the proteophosphoglycan is secreted in large amounts by amastigotes *via* their flagellar pockets into the parasitophorous vacuoles of host cells. It is thought that these molecules protect the amastigotes inside these vacuoles (51).

Moreover, N-linked glycans are also thought to be involved in *Leishmania* virulence. One of the most significant purposes of Asn-linked glycans is that they are required for the right folding of polypeptides in the endoplasmic reticulum, this folding is important to transport manufactured proteins to their final destination. A study has used tunicamycin to reveal the importance of these molecules in *Leishmnai* parasite (57). Tunicamycin is a specific inhibitor of N-glycan biosynthesis. It was observed that tunicamycin-resistant Leishmania lose their virulence in culture more slowly than their non-resistant companions and they showed a high degree of virulence in experimental mice. They also infected macrophages *in vitro* more efficiently. The ability of the tunicamycin-resistant cells to overcome the inhibitory effect of tunicamycin was resulted from a high level of the glycosyltransferase enzyme that regulates N glycosylation of leishmanial proteins essential for *Leishmania* to establish intracellular parasitism (58).

An amastigote stage-specific protein termed A2 was first discovered in *L. donovani* and designated as a virulence determinant is *Leishmania sp* (59). It is isolated from subtractive cDNA hybridization libraries as a family of amastigotes specific transcripts of 45-100 kDa proteins encoded by at least 7 genes. These proteins are repetitive sequences (40 to >90 repeats), each contains a secretory leader sequence and 10 amino acids sequence. A2 proteins present mainly in the cytoplasm of the amastigotes and almost absent in the promastigote because more than 90% of serum from visceral leishmaniasis objects contain anti-A2 antibodies. A2 deficient *L. donovani* amastigotes were created by antisense RNA.The resulting mutants were viable in culture but showed a reduced ability to multiply in cultured macrophages. Their virulence in mice was considerably affected and the amastigotes that survived in mice has restored their A2 expression (60). Interestingly, A2 is absent in the genome of *L. major* and *L. tropica* but present in all other *Leishmania* species involving *L. donovani, L.chagasi, and L. infantum.* More interestingly, L. major has non-expressed A2 pseudogenes due to absence of the various repeats in the protein multiple sections of the genome. In depth genetic examination of DNA sequence and gene regulation in *L. major* and *L. donovani* have revealed that phenotypically distinct species have genotypic differences (61).

Studies have revealed that restoring amastigote –specific A2 expression in *L. major* has changed the resulting phenotype of this cutaneous parasite. The *L. major* parasite was not able to cause cutaneous infection in susceptible BALB/c or resistant C57BL6 mice. Also, it had unexpected capability to travel out of the ear dermis, relative to control *L. major.* This phenotype is similar to *L. donovani.* Migration of the parasite to the liver was also observed. Another study has revealed that restoring the A2 expression in *L. major* and infecting BALB/c mice through tail vein injection resulted in splenomegaly, a phenotype typical to *L. donovani* (60, 61). Surprisingly, karyotype analyses in L. mexicana complex (L. mexicana and L. amazonensis) have shown the presence of the A2 coding sequences. This was also supported by Western blot analysis that indicated the presence of three large proteins of > 200 kDa in *L. Mexicana* (62). Although A2 is present in *L. mexicana* complex, these parasites are related to diffuse cutaneous leishmaniasis, but not visceral leishmaniasis. Moreover, there are some visceral leishmaniasis cases reported due to *L. tropica*, a causative agent of cutaneous infection, in some soldiers of Operation Desert Storm during the gulf war in 1990. This systemic illness was given the name “viscerotropic” leishmaniasis to discriminate it from “visceral” leishmaniasis. Accordingly, A2 is not the only responsible factor of visceral leishmaniasis. But in the case of *L. donovani*, it is very critical as a virulence factor causing the visceral infection. Studies have revealed that that immunization with A2, as protein or DNA, protects against *L. donovani* infection, this has been used widely in the field of vaccine development against visceral leishmaniasis (63). In addition, another study has examined the significant defensive outcome of immunization with the recombinant A2 (rA2) proteins against *L. amazonensis* contagion. Protection was linked with the favored and constant induction of a Th1 immune reaction (64–66).

### Metabolic Changes of Host With *Leishmania* and Its Survival

*Leishmania*’s manipulation of host metabolic fluxes is a strategy for circumventing the host immune response, resulting in long-term parasite survival and playing a key role in infection pathology. Specific Leishmania-induced metabolic changes in infected macrophages have been linked to infection resistance or susceptibility. As a result, understanding the multilayer relationships between metabolism and function on innate immune cells during infection has a lot of therapeutic or preventive potential.

In recent years, methods and technology for detecting, identifying, and measuring metabolites within a cell and its surroundings with high sensitivity have vastly improved, spawning the flourishing subject of metabolomics. They may now be used in research on disease agents such as parasites, which helps to better understand their biology while also allowing for better drug discovery, illness diagnostics, and therapy (67, 68). Too far, several research on *Leishmania* metabolites have been published, offering both precise methods that may be used and insights into the biochemistry and mechanisms of drug resistance in each species (69). It is widely known that one way by which some infections reduce the immune response of their mammalian hosts is by the depletion of amino acids essential to immunological processes (70).

Macrophages probably play a critical role in the Leishmania parasite, both historically and clinically, diagnostically, and immunologically. The first histological account of the pathophysiology of cutaneous leishmaniasis (CL; called “sart sore” in his country) was published in 1898 by Russian-born military doctor Peter Borovsky (1863–1932) from Taschkent, who described the intimate alliance between macrophages and Leishmania. He not only correctly identified the underlying infectious agent as a protozoan parasite, but he also recognized and graphically illustrated its size (on average 1.5 to 2 m) and localization within host cells, which he referred to as “lymphoid and epithelioid cells” because he was presumably unaware of Metschniko’s characterization of macrophages (71). The microscopical detection of oval-shaped Leishmania amastigotes within tissue macrophages (i.e. histiocytes) of cutaneous, splenic, hepatic, or bone marrow biopsies (with the typical disc formed kinetoplast adjacent to the flagellar basal body) is still a central pillar of the microbiological diagnosis of both cutaneous and visceral leishmaniasis.

Activation of macrophages from permissive host cells to leishmanicidal effector cells during Leishmania infection is dependent on cytokines, particularly IFN-, which is produced by a variety of cell types (e.g., natural killer [NK] cells, CD4+ or CD8+ T cells, and certain types of NKT cells) and is already released during the early stages of infection (72, 73).

The immunological concept for controlling intracellular Leishmania amastigotes includes a number of components such as reactive oxygen and nitrogen species (ROS and RNS), the impact of microenvironmental and metabolic parameters, and other antileishmanial effectors. Because of their expression of MHC class II and costimulatory molecules, presentation of antigens, secretion of cytokines, and release of RNS and ROS during the innate and acute phases of Leishmania infections, macrophages not only serve as host cells and antileishmanial effector cells, but also as immunoregulatory cells. Infection with Leishmania can alter these processes in either a good or negative way, depending on the parasite species, developmental stage, and experimental setup (74).

Leishmania amastigotes are highly reliant on external supplies of amino acids, which are controlled by the nutrient-sensing pathways previously reported (75, 76). While the defensive response to viruses is heavily reliant on amino acid metabolism, diseases can manipulate this metabolism as a means of spreading throughout the host. The key amino acids arginine, tryptophan, and glutamine are important in immunological control and nutritional competition between the host and pathogens (77). A metabolomic investigation of L. amazonensis-infected macrophages revealed an increase in L-arginine metabolism toward polyamine synthesis, enhancing the intracellular redox balance of infected cells and protecting the parasites from NO and ROS from the host (78). Increased IL-10 production by infected macrophages corresponds with increased Arg-1 activity during Leishmania infection, forming a positive feedback loop that enhances Arg-1 activity (79). The regulation of visceral leishmaniasis relies heavily on glutamine metabolism, and Leishmania amastigotes rely heavily on mitochondrial metabolism for *de novo* glutamate and glutamine synthesis (80). Glutamine synthetase (GS) is a protein that produces glutamine from glutamate and ammonia, and it has been found in both promastigote and amastigote Leishmania parasites (81). The availability of tryptophan was also discovered to be critical for Leish mania development inside macrophages. Tryptophan depletion caused by idoleamine-2,3-dioxygenase (IDO) activation, a kynurenine pathway enzyme, represents a key antibacterial mechanism during Leishmania infection by lowering tryptophan availability to intracellular amastigotes (82). During infection, tryptophan 2,3-deoxygenase (TDO) was found to compensate for IDO. TDO is identified as a limitation factor in human skin lesions during CL, indicating that its expression may govern parasite growth in lesions, and pharmacological suppression of TDO enhanced parasite load in *ex vivo* Leishmania major-infected macrophages (82). The research demonstrates the importance of IDO and TDO as pathogen growth regulators, either by dampening host immunity or by influencing infection progression by reducing pathogen growth.

Intracellular Leishmania survival is further influenced by the host’s glycolytic and lipid metabolism. Early after *in vitro* infection, Leishmania-infected macrophages upregulate the transcription of numerous glycolytic genes (e.g. hexokin pyruvate kinase isozymes M2, lactate dehydrogenase A), which correlates with intracellular parasite survival (83–86). Infected macrophages were reported to have less intracellular amastigotes when glycolysis was inhibited with 2-deoxyglucose (2-DG) (86). In addition, Leishmania infection reduces the sensitivity of mitochondrial membrane permeabilization to apoptotic stimuli, implying a relationship between mitochondria and parasite persistence (82). Overall, glycolysis was found to be crucial in the early stages of Leishmania spp. infection in macrophages and neutrophils, whereas enhanced mitochondrial metabolism was revealed to be important in the late stages of infection (87, 88).

We can deduce from these data that metabolic reprogramming of Leishmania-infected macrophages is a driving factor for Leishmania parasite infection and immune evasion by reducing the ability of infected cells to elicit robust immunological responses. Thus, regulating the host nutrient-sensing pathways (AMPK, mTOR, and HIF-1a), which affects amino acid, cholesterol, and fatty acid metabolism, appears to be a crucial regulator of Leishmania infection, while the molecular mechanism behind such alterations is unknown. Overall, the evidence presented here suggests that modulating host metabolism during infection could be a promising treatment approach for leishmaniasis (74).

## Conclusion

To conclude, *Leishmania* parasites have a variety of invasive/evasive and pathoantigenic factors that appear to be relevant for *Leishmania* infection of macrophages and intracellular parasitism. These determinants are known as “virulence factors” and are thought to be ideal targets for designing particular inhibitors to decrease *Leishmania* sp (64). virulence through gene mutations, resulting in infectious but non-pathogenic mutants for vaccine immunization. Hence, biosynthetic pathways are essential for the survival of any parasite and for the production of anti-parasitic drugs that target enzymes involved in parasite establishment. As a result, parasite infection is kept at a low level, and in some situations, it is used as a weapon to totally eradicate infection. Future research on the virulence factors of distinct Leishmania species could aid in the development of a novel vaccine to treat the disease by providing a better understanding of the disease’s etiology.

## Author Contributions

The author confirms being the sole contributor of this work and has approved it for publication.

## Conflict of Interest

The author declares that the research was conducted in the absence of any commercial or financial relationships that could be construed as a potential conflict of interest.

## Publisher’s Note

All claims expressed in this article are solely those of the authors and do not necessarily represent those of their affiliated organizations, or those of the publisher, the editors and the reviewers. Any product that may be evaluated in this article, or claim that may be made by its manufacturer, is not guaranteed or endorsed by the publisher.

## References

[1] Cardoso T Bezerra C Medina LS Ramasawmy R Scheriefer A Bacellar O. Leishmania Braziliensis Isolated From Disseminated Leishmaniasis Patients Downmodulate Neutrophil Function. Parasite Immunol (2019) 41(5):e12620. doi: 10.1111/pim.12620 30815888 PMC6519172

[2] Gomez EA Kato H Torres-Romero EX Velez LN Villegas NV Martillo VP. Leishmaniasis Caused by Leishmania (Viannia) Guyanensis in North-Central Pacific Region of Ecuador: A Clinico-Epidemiological Feature. Acta Tropica (2018) 185:204–11. doi: 10.1016/j.actatropica.2018.05.016 29852129

[3] Ismail A El Hassan AM Kemp K Gasim S Kadaru AEGMY Møller T. Immunopathology of Post Kala-Azar Dermal Leishmaniasis (PKDL): T-Cell Phenotypes and Cytokine Profile. J Pathol (1999) 189(4):615–22. doi: 10.1002/(SICI)1096-9896(199912)189:4<615::AID-PATH466>3.0.CO;2-Z 10629566

[4] Gasim S Elhassan AM Kharazmi A Khalil EAG Ismail A Theander TG. The Development of Post-Kala-Azar Dermal Leishmaniasis (PKDL) is Associated With Acquisition of Leishmania Reactivity by Peripheral Blood Mononuclear Cells (PBMC). Clin Exp Immunol (2000) 119(3):523–9. doi: 10.1046/j.1365-2249.2000.01163.x PMC1905576 10691926

[5] Jain V Jain K. Molecular Targets and Pathways for the Treatment of Visceral Leishmaniasis. Drug Discovery Today (2018) 23(1):161–70. doi: 10.1016/j.drudis.2017.09.006 28919438

[6] Kaye PM Cruz I Picado A Van Bocxlaer K Croft SL. Leishmaniasis Immunopathology—Impact on Design and Use of Vaccines, Diagnostics and Drugs. Semin Immunopathol (2020) 42:(3):247–64. doi: 10.1007/s00281-020-00788-y 32152715

[7] Khan MI Mishra A Jha PK Abhishek K Chaba R Das P. DNA Polymerase β of Leishmania Donovani is Important for Infectivity and it Protects the Parasite Against Oxidative Damage. Int J Biol Macromol (2019) 124:291–303. doi: 10.1016/j.ijbiomac.2018.11.159 30452983

[8] Aoki JI Laranjeira-Silva MF Muxel SM Floeter-Winter LM. The Impact of Arginase Activity on Virulence Factors of Leishmania Amazonensis. Curr Opin Microbiol (2019) 52:110–5. doi: 10.1016/j.mib.2019.06.003 31306995

[9] Qi H Popov V Soong L. Leishmania Amazonensis Dendritic Cell Interactions *In Vitro* and the Priming of Parasite-Specific CD4+ T Cells In Vivo. J Immunol (2001) 167(8):4534–42. doi: 10.4049/jimmunol.167.8.4534 11591781

[10] Al-Khalaifah H Al-Nasser A. Immune Response of Molluscs. Molluscs: IntechOpen (2018) p:1. doi: 10.5772/intechopen.81778

[11] Elmahallawy EK Alkhaldi AA. Insights Into Leishmania Molecules and Their Potential Contribution to the Virulence of the Parasite. Veterinary Sci (2021) 8(2):33. doi: 10.3390/vetsci8020033 PMC7924612 33672776

[12] Castro H Rocha MI Silva R Oliveira F Gomes-Alves AG Cruz T. Functional Insight Into the Glycosomal Peroxiredoxin of Leishmania. Acta Tropica (2020) 201:105217. doi: 10.1016/j.actatropica.2019.105217 31605692

[13] Battista T Colotti G Ilari A Fiorillo AJM. Targeting Trypanothione Reductase, a Key Enzyme in the Redox Trypanosomatid Metabolism, to Develop New Drugs Against Leishmaniasis and Trypanosomiases. Molecules (2020) 25(8):1924. doi: 10.3390/molecules25081924 PMC7221613 32326257

[14] Suman SS Amit A Singh KP Gupta P Equbal A Kumari A. Cytosolic Tryparedoxin of Leishmania Donovani Modulates Host Immune Response in Visceral Leishmaniasis. Cytokine (2018) 108:1–8. doi: 10.1016/j.cyto.2018.03.010 29554571

[15] Tunes LG Morato RE Garcia A Schmitz V Steindel M Corrêa-Junior JD. Preclinical Gold Complexes as Oral Drug Candidates to Treat Leishmaniasis Are Potent Trypanothione Reductase Inhibitors. ACS Infect Dis (2020) 6(5):1121–39. doi: 10.1021/acsinfecdis.9b00505 32283915

[16] Prive C Descoteaux A. Leishmania Donovani Promastigotes Evade the Activation of Mitogen-Activated Protein Kinases P38, C-Jun N-Terminal Kinase, and Extracellular Signal-Regulated Kinase-1/2 During Infection of Naive Macrophages. Eur J Immunol (2000) 30(8):2235–44. doi: 10.1002/1521-4141(2000)30:8<2235::AID-IMMU2235>3.0.CO;2-9 10940915

[17] Mahami-Oskouei M Mohebali M Spotin A Alizadeh Z. A Review of Effectual Factors in the Pathogenesis of Leishmania Parasites. J Ardabil Univ Med Sci (2018) 18(3):279–97. doi: 10.29252/jarums.18.3.279

[18] Abu-Rezq TS James CM. Beneficial Effects of Using Commercial Probiotics for Producing Rotifers for Aquaculture. J Aquacult Tropics (2005) 21:1–11.

[19] Dermine J-F Scianimanico S Privé C Descoteaux A Desjardins M. Leishmania Promastigotes Require Lipophosphoglycan to Actively Modulate the Fusion Properties of Phagosomes at an Early Step of Phagocytosis. Cell Microbiol (2000) 2(2):115–26. doi: 10.1046/j.1462-5822.2000.00037.x 11207568

[20] de Carvalho RVH Andrade WA Lima-Junior DS Dilucca M de Oliveira CV Wang K. Leishmania Lipophosphoglycan Triggers Caspase-11 and the Non-Canonical Activation of the NLRP3 Inflammasome. Cell Rep (2019) 26(2):429–37.e5. doi: 10.1016/j.celrep.2018.12.047 30625325 PMC8022207

[21] Turco SJ Späth GF Beverley SM. Is Lipophosphoglycan a Virulence Factor? A Surprising Diversity Between Leishmania Species. Trends Parasitol (2001) 17(5):223–6. doi: 10.1016/S1471-4922(01)01895-5 11323305

[22] Belen Carrillo M Gao W Herrera M Alroy J Moore JB Beverley SM. Heterologous Expression of Trypanosoma Cruzi Trans Sialidase in Leishmania Major Enhances Virulence. Infect Immun (2000) 68(5):2728–34. doi: 10.1128/IAI.68.5.2728-2734.2000 PMC97481 10768966

[23] Holm Å Tejle K Gunnarsson T Magnusson KE Descoteaux A Rasmusson B. Role of Protein Kinase C α for Uptake of Unopsonized Prey and Phagosomal Maturation in Macrophages. Biochem Biophys Res Commun (2003) 302(4):653–8. doi: 10.1016/S0006-291X(03)00231-6 12646218

[24] Thomas I Monika D Harbecke D. Phosphoglycan Repeat-Deficient Leishmania Mexicana Parasites Remain Infectious to Macrophages and Mice. J Biol Chem (2001) 276(7):4988–97. doi: 10.1074/jbc.M008030200 11071892

[25] Ilg T. Lipophosphoglycan is Not Required for Infection of Macrophages or Mice by Leishmania Mexicana. EMBO J (2000) 19(9):1953–62. doi: 10.1093/emboj/19.9.1953 PMC305689 10790362

[26] Bahr V Stierhof Y-D Ilg T Demar M Quinten M Overath PJM. Expression of Lipophosphoglycan, High-Molecular Weight Phosphoglycan and Glycoprotein 63 in Promastigotes and Amastigotes of Leishmania Mexicana. Mol Biochem Parasitol (1993) 58(1):107–21. doi: 10.1016/0166-6851(93)90095-F 8459823

[27] Ramamoorthy R Donelson J Paetz K Maybodi M Roberts S Wilson M. Three Distinct RNAs for the Surface Protease Gp63 are Differentially Expressed During Development of Leishmania Donovani Chagasi Promastigotes to an Infectious Form. J Biolog Chem (1992) 267(3):1888–95. doi: 10.1016/S0021-9258(18)46030-9 1370484

[28] Macdonald MH Morrison CJ McMaster WR. Analysis of the Active Site and Activation Mechanism of the Leishmania Surface Metalloproteinase GP63. Biochim Biophys Acta (BBA) Protein Structure Mol Enzymol (1995) 1253(2):199–207. doi: 10.1016/0167-4838(95)00155-5 8519803

[29] Yao C Donelson JE Wilson ME. The Major Surface Protease (MSP or GP63) of Leishmania Sp. Biosynthesis, regulation of expression, and function. Mol Biochem Parasitol (2003) 132(1):1–16. doi: 10.1016/S0166-6851(03)00211-1 14563532

[30] Wozencraft AO Blackwell JM. Increased Infectivity of Stationary-Phase Promastigotes of Leishmania Donovani: Correlation With Enhanced C3 Binding Capacity and CR3-Mediated Attachment to Host Macrophages. Immunology (1987) 60(4):559–63. PMC1453286 2953670

[31] Corradin S Ransijn A Corradin G Bouvier J Delgado MB Fernandez-Carneado J. Novel Peptide Inhibitors of Leishmania Gp63 Based on the Cleavage Site of MARCKS (Myristoylated Alanine-Rich C Kinase Substrate)-Related Protein. Biochem J (2002) 367(3):761–9. doi: 10.1042/bj20020386 PMC1222923 12137567

[32] McGwire BS Chang K-P Engman DM. Migration Through the Extracellular Matrix by the Parasitic Protozoan Leishmania Is Enhanced by Surface Metalloprotease Gp63. Infect Immun (2003) 71(2):1008–10. doi: 10.1128/IAI.71.2.1008-1010.2003 PMC145380 12540585

[33] Joshi PB Kelly BL Kamhawi S Sacks DL McMaster WR. Targeted Gene Deletion in Leishmania Major Identifies Leishmanolysin (GP63) as a Virulence Factor. Mol Biochem Parasitol (2002) 120(1):33–40. doi: 10.1016/S0166-6851(01)00432-7 11849703

[34] Spath G Epstein L Leader B Singer S Avila H Turco S. Lipophosphoglycan is a Virulence Factor Distinct From Related Glycoconjugates in the Protozoan Parasite Leishmania Major. Proc Natl Acad Sci (2000) 97(16):9258–63. doi: 10.1073/pnas.160257897 PMC16855 10908670

[35] Jesus-Santos FH Lobo-Silva J Ramos PIP Descoteaux A Lima JB Borges VM. LPG2 Gene Duplication in Leishmania Infantum: A Case for CRISPR-Cas9 Gene Editing. Front Cell Infect Microbiol (2020) 408. doi: 10.3389/fcimb.2020.00408 PMC7438834 32903718

[36] Sajid M McKerrow JH. Cysteine Proteases of Parasitic Organisms. Mol Biochem Parasitol (2002) 120(1):1–21. doi: 10.1016/S0166-6851(01)00438-8 11849701

[37] Mottram JC Souza AE Hutchison JE Carter R Frame MJ Coombs GH. Evidence From Disruption of the Lmcpb Gene Array of Leishmania Mexicana That Cysteine Proteinases are Virulence Factors. Proc Natl Acad Sci U S A (1996) 93(12):6008–13. doi: 10.1073/pnas.93.12.6008 PMC39179 8650210

[38] Alexander J Coombs GH Mottram JC. Leishmania Mexicana Cysteine Proteinase-Deficient Mutants Have Attenuated Virulence for Mice and Potentiate a Th1 Response. J Immunol (1998) 161(12):6794–801. 9862710

[39] Svensjo E Cyrino FZ Juliano L Scharfstein J. Plasma Leakage Induced in Postcapillary Venules by the Major Cysteine- Proteinase From Trypanosoma Cruzi and its Modulation by H1-Blocker Mepyramine. Microvasc Res (1997) 54(1):93. doi: 10.1006/mvre.1997.2020 9245650

[40] Padilla A Noiva R Lee N Mohan KVK Nakhasi HL Debrabant A. An Atypical Protein Disulfide Isomerase From the Protozoan Parasite Leishmania Containing a Single Thioredoxin-Like Domain. J Biol Chem (2003) 278(3):1872–8. doi: 10.1074/jbc.M210322200 12427741

[41] Wiese M. A Mitogen-Activated Protein (MAP) Kinase Homologue of Leishmania Mexicana Is Essential for Parasite Survival in the Infected Host. EMBO J (1998) 17(9):2619–28. doi: 10.1093/emboj/17.9.2619 PMC1170603 9564044

[42] McConville MJ Ferguson MJBJ. The Structure, Biosynthesis and Function of Glycosylated Phosphatidylinositols in the Parasitic Protozoa and Higher Eukaryotes. Biochem J (1993) 294(Pt 2):305. doi: 10.1042/bj2940305 8373346 PMC1134455

[43] Turco SJ Descoteaux A. The Lipophosphoglycan of Leishmania Parasites. Ann Rev Microbiol (1992) 46(1):65–92. doi: 10.1146/annurev.mi.46.100192.000433 1444269

[44] McConville MJ Blackwell JM. Developmental Changes in the Glycosylated Phosphatidylinositols of Leishmania Donovani. Characterization of the Promastigote and Amastigote Glycolipids. J Biolog Chem (1991) 266(23):15170–9. doi; 10.1016/S0021-9258(18)98600-X 1831200

[45] McConville MJ Collidge T Ferguson M Schneider PJ. The Glycoinositol Phospholipids of Leishmania Mexicana Promastigotes. Evidence for the Presence of Three Distinct Pathways of Glycolipid Biosynthesis. J Biolog Chem (1993) 268(21):15595–604. doi: 10.1016/S0021-9258(18)82298-0 8340385

[46] Ilgoutz SC Zawadzki JL Ralton JE McConville MJ. Evidence That Free GPI Glycolipids are Essential for Growth of Leishmania Mexicana. EMBO J (1999) 18(10):2746–55. doi: 10.1093/emboj/18.10.2746 PMC1171356 10329621

[47] Ma D Russell DG Beverley SM Turco SJ. Golgi GDP-Mannose Uptake Requires Leishmania LPG2 A Member of a Eukaryotic Family of Putative Nucleotide-Sugar Transporters. J Biological Chem (1997) 272(6):3799–805. doi: 10.1074/jbc.272.6.3799 9013638

[48] Tachado SD Gerold P Schwarz R Novakovic S McConville M Schofield L. Signal Transduction in Macrophages by Glycosylphosphatidylinositols of Plasmodium, Trypanosoma, and Leishmania: Activation of Protein Tyrosine Kinases and Protein Kinase C by Inositolglycan and Diacylglycerol Moieties. Proc Nat Acad Sci (1997) 94(8):4022–7. doi: 10.1073/pnas.94.8.4022 PMC20561 9108098

[49] Winter G Fuchs M McConville MJ Stierhof Y-D Overath P. Surface Antigens of Leishmania Mexicana Amastigotes: Characterization of Glycoinositol Phospholipids and a Macrophage-Derived Glycosphingolipid. J Cell Sci (1994) 107(9):2471–82. doi: 10.1242/jcs.107.9.2471 7844164

[50] Lovelace JK Gottlieb M. Comparison of Extracellular Acid Phosphatases From Various Isolates of Leishmania. JTAjotm Hygiene (1986) 35(6):1121–8. doi: 10.4269/ajtmh.1986.35.1121 3789268

[51] Ilg T Stierhof Y McConville M Overath P. Purification, Partial Characterization and Immunolocalization of a Proteophosphoglycan Secreted by Leishmania Mexicana Amastigotes. Eur J Cell Biol (1995) 66(2):205–15. 7774606

[52] Ilg T Overath P Ferguson M Rutherford T Campbell DG McConville M. O-And N-Glycosylation of the Leishmania Mexicana-Secreted Acid Phosphatase. Characterization of a New Class of Phosphoserine-Linked Glycans. J Biolog Chem (1994) 269(39):24073–81. doi: 10.1016/S0021-9258(19)51049-3 7929059

[53] Landfear SM Ignatushchenko M. The Flagellum and Flagellar Pocket of Trypanosomatids. Mol Biochem Parasitol (2001) 115(1):1–17. doi: 10.1016/S0166-6851(01)00262-6 11377735

[54] Treisman R. Regulation of Transcription by MAP Kinase Cascades. Current Opin Cell Biol (1996) 8(2):205–15. doi: 10.1016/S0955-0674(96)80067-6 8791420

[55] Stierhof Y-D Bates PA Jacobson RL Rogers ME Schlein Y Handman E. Filamentous Proteophosphoglycan Secreted by Leishmania Promastigotes Forms Gel-Like Three-Dimensional Networks That Obstruct the Digestive Tract of Infected Sandfly Vectors. Eur J Cell Biol (1999) 78(10):675–89. doi: 10.1016/S0171-9335(99)80036-3 10569240

[56] Ilg T Montgomery J Stierhof Y-D Handman E. Molecular Cloning and Characterization of a Novel Repeat-Containing Leishmania Major Gene, Ppg1, That Encodes a Membrane-Associated Form of Proteophosphoglycan With a Putative Glycosylphosphatidylinositol Anchor. J Biolog Chem (1999) 274(44):31410–20. doi: 10.1074/jbc.274.44.31410 10531342

[57] Koizumi N Ujino T Sano H Chrispeels M. Overexpression of a Gene That Encodes the First Enzyme in the Biosynthesis of Asparagine-Linked Glycans Makes Plants Resistant to Tunicamycin and Obviates the Tunicamycin-Induced Unfolded Protein Response. Plant Physiol (1999) 121(2):353–62. doi: 10.1104/pp.121.2.353 PMC59397 10517826

[58] Kink JA Chang K. Biological and Biochemical Characterization of Tunicamycin-Resistant Leishmania Mexicana: Mechanism of Drug Resistance and Virulence. JI Immun (1987) 55(7):1692–700. doi: 10.1128/iai.55.7.1692-1700.1987 PMC260580 3036710

[59] Charest H Matlashewski G. Developmental Gene Expression in Leishmania Donovani: Differential Cloning and Analysis of an Amastigote-Stage-Specific Gene. JM Biol C (1994) 14(5):2975–84. doi: 10.1128/mcb.14.5.2975-2984.1994 PMC358665 7545921

[60] Zhang W Matlashewski G. Loss of Virulence in Leishmania Donovani Deficient in an Amastigote-Specific Protein, A2. Microbiology (1997) 94:8807–11. doi: 10.1073/pnas.94.16.8807 PMC23140 9238059

[61] Zhang W-W Mendez S Ghosh A Myler P Ivens A Clos J. Comparison of the A2 Gene Locus in Leishmania Donovani and Leishmania Major and Its Control Over Cutaneous Infection. J Biol Chem (2003) 278(37):35508–15. doi: 10.1074/jbc.M305030200 12829719

[62] Ghedin E Zhang WW Charest H Sundar S Kenney RT Matlashewski G. Antibody Response Against a Leishmania Donovani Amastigote-Stage- Specific Protein in Patients With Visceral Leishmaniasis. Clin Diagn Lab Immunol (1997) 4(5):530–5. doi: 10.1128/cdli.4.5.530-535.1997 PMC170587 9302200

[63] Ghosh A Zhang WW Matlashewski G. Immunization With A2 Protein Results in a Mixed Th1/Th2 and a Humoral Response Which Protects Mice Against Leishmania Donovani Infections. Vaccine (2001) 20(1):59–66. doi: 10.1016/S0264-410X(01)00322-X 11567746

[64] Carvalho FAA Charest H Tavares CAP Matlashewski G Valente EP Rabello A. Diagnosis of American Visceral Leishmaniasis in Humans and Dogs Using the Recombinant Leishmania Donovani A2 Antigen. Diagn Microbiol Infect Dis (2002) 43(4):289–95. doi: 10.1016/S0732-8893(02)00410-8 12151189

[65] Coelho EAF Tavares CAP Carvalho FAA Chaves KF Teixeira KN Rodrigues RC. Immune Responses Induced by the Leishmania (Leishmania) Donovani A2 Antigen, But Not by the LACK Antigen, Are Protective Against Experimental Leishmania (Leishmania) Amazonensis Infection. Infect Immun (2003) 71(7):3988–94. doi: 10.1128/IAI.71.7.3988-3994.2003 PMC162020 12819086

[66] Mougneau E Altare F Wakil AE Zheng S Coppola T Wang ZE. Expression Cloning of a Protective Leishmania Antigen. Science (1995) 268:563–56. doi: 10.1126/science.7725103 7725103

[67] Hurrell BP Regli IB Tacchini-Cottier F. Different Leishmania Species Drive Distinct Neutrophil Functions. Trends Parasitol (2016) 32(5):392–401. doi: 10.1016/j.pt.2016.02.003 26944469

[68] von Stebut E Tenzer S. Cutaneous Leishmaniasis: Distinct Functions of Dendritic Cells and Macrophages in the Interaction of the Host Immune System With Leishmania Major. Int J Med Microbiol (2018) 308(1):206–14. doi: 10.1016/j.ijmm.2017.11.002 29129568

[69] Bates PA. Transmission of Leishmania Metacyclic Promastigotes by Phlebotomine Sand Flies. Int J Parasitol (2007) 37(10):1097–106. doi: 10.1016/j.ijpara.2007.04.003 PMC2675784 17517415

[70] Carter CR Whitcomb JP Campbell JA Mukbel RM McDowell MA. Complement Receptor 3 Deficiency Influences Lesion Progression During Leishmania Major Infection in BALB/c Mice. Infect Immun (2009) 77(12):5668–75. doi: 10.1128/IAI.00802-08 PMC2786468 19797068

[71] Hoare CA. Early Discoveries Regarding the Parasite of Oriental Sore. Trans R Soc Trop Med Hygiene (1938) 32(1):66–92. doi: 10.1016/S0035-9203(38)90097-5

[72] Mcgavin MJ Arsic B Nickerson NN. Evolutionary Blueprint for Host-and Niche-Adaptation in Staphylococcus Aureus Clonal Complex CC30. Front Cell Infect Microbiol (2012) 2:48. doi: 10.3389/fcimb.2012.00048 22919639 PMC3417553

[73] Prajeeth CK Haeberlein S Sebald H Schleicher U Bogdan C. Leishmania-Infected Macrophages are Targets of NK Cell-Derived Cytokines But Not of NK Cell Cytotoxicity. Infect Immun (2011) 79(7):2699–708. doi: 10.1128/IAI.00079-11 PMC3191990 21518784

[74] Bogdan C. Macrophages as Host, Effector and Immunoregulatory Cells in Leishmaniasis: Impact of Tissue Micro-Environment and Metabolism. Cytokine: X (2020) 2(4):100041. doi: 10.1016/j.cytox.2020.100041 33604563 PMC7885870

[75] Bar-Peled L Sabatini DM. Regulation of Mtorc1 by Amino Acids. Trends Cell Biol (2014) 24(7):400–6. doi: 10.1016/j.tcb.2014.03.003 PMC4074565 24698685

[76] Dalle Pezze P Ruf S Sonntag AG Langelaar-Makkinje M Hall P Heberle AM. A Systems Study Reveals Concurrent Activation of AMPK and mTOR by Amino Acids. Nat Commun (2016) 7(1):1–19. 10.1038/ncomms13254 PMC5121333 27869123

[77] Ren W Rajendran R Zhao Y Tan B Wu G Bazer FW. Amino Acids as Mediators of Metabolic Cross Talk Between Host and Pathogen. Front Immunol (2018) 9:319. doi: 10.3389/fimmu.2018.00319 29535717 PMC5835074

[78] Muxel SM Mamani-Huanca M Aoki JI Zampieri RA Floeter-Winter LM López-Gonzálvez Á. Metabolomic Profile of BALB/c Macrophages Infected With Leishmania Amazonensis: Deciphering L-Arginine Metabolism. Int J Mol Sci (2019) 20(24):6248. doi: 10.3390/ijms20246248 PMC6940984 31835767

[79] Mandal A Das S Kumar A Roy S Verma S Ghosh AK. L-Arginine Uptake by Cationic Amino Acid Transporter Promotes Intra-Macrophage Survival of Leishmania Donovani by Enhancing Arginase-Mediated Polyamine Synthesis. Front Immunol (2017) 8:839. doi: 10.3389/fimmu.2017.00839 28798743 PMC5526900

[80] Saunders EC Ng WW Kloehn J Chambers JM Ng M McConville MJ. Induction of a Stringent Metabolic Response in Intracellular Stages of Leishmania Mexicana Leads to Increased Dependence on Mitochondrial Metabolism. PloS Pathogens (2014) 10(1):e1003888. doi: 10.1371/journal.ppat.1003888 24465208 PMC3900632

[81] Kumar V Yadav S Soumya N Kumar R Babu NK Singh S. Biochemical and Inhibition Studies of Glutamine Synthetase From Leishmania Donovani. Microbial Pathogenesis (2017) 107:164–74. doi: 10.1016/j.micpath.2017.03.024 28351708

[82] Rodrigues V André S Maksouri H Mouttaki T Chiheb S Riyad M. Transcriptional Analysis of Human Skin Lesions Identifies Tryptophan-2, 3-Deoxygenase as a Restriction Factor for Cutaneous Leishmania. Frontiers in Cellular and Infection Microbiology. Front Cell Inf Microbiol (2019) 338:338. doi: 10.3389/fcimb.2019.00338 PMC6788307 31637219

[83] Moreira D Rodrigues V Abengozar M Rivas L Rial E Laforge M. Leishmania Infantum Modulates Host Macrophage Mitochondrial Metabolism by Hijacking the SIRT1-AMPK Axis. PloS Pathogens (2015) 11(3):e1004684. doi: 10.1371/journal.ppat.1004684 25738568 PMC4349736

[84] Rabhi I Rabhi S Ben-Othman R Rasche A Consortium S Daskalaki A. Transcriptomic Signature of Leishmania Infected Mice Macrophages: A Metabolic Point of View. PLoS Negl Trop Dis (2012) 6(8):e1763. doi: 10.1371/journal.pntd.0001763 22928052 PMC3424254

[85] Rabhi S Rabhi I Trentin B Piquemal D Regnault B Goyard S. Lipid Droplet Formation, Their Localization and Dynamics During Leishmania Major Macrophage Infection. PloS One (2016) 11(2):e0148640. doi: 10.1371/journal.pone.0148640 26871576 PMC4752496

[86] Basu M Gupta P Dutta A Jana K Ukil A. Increased Host ATP Efflux and its Conversion to Extracellular Adenosine is Crucial for Establishing Leishmania Infection. J Cell Sci (2020) ;133(7):jcs239939. doi: 10.1242/jcs.239939 32079656

[87] Karthik L Kumar G Keswani T Bhattacharyya A Chandar SS Bhaskara Rao K. Protease Inhibitors From Marine Actinobacteria as a Potential Source for Antimalarial Compound. PloS One (2014) 9(3):e90972. doi: 10.1371/journal.pone.0090972 24618707 PMC3949715

[88] Zhang Q Meng Y Wang K Zhang X Chen W Sheng J. Inflammation and Antiviral Immune Response Associated With Severe Progression of COVID-19. Front Immunol (2021) 12:135. doi: 10.3389/fimmu.2021.631226 PMC7930228 33679778

